# Rapid and Ultrasensitive Detection of Dioctyltin in Textiles Using Surface-Enhanced Raman Spectroscopy (SERS): Mechanistic Insights and Practical Applications

**DOI:** 10.3390/s26061891

**Published:** 2026-03-17

**Authors:** Zheyu Shen, Qiang Chen, Xia Gao, Yan Liu, Jiamin Wang, Pei Liang

**Affiliations:** 1 College of Metrology Measurement and Instrument, China Jiliang University, Hangzhou 310018, China; p23020854078@cjlu.edu.cn; 2 College of Energy Environment and Safety Engineering, China Jiliang University, Hangzhou 310018, China; 3 College of Optical and Electronic Technology, China Jiliang University, Hangzhou 310018, China; 4 Institute of Analysis and Testing, Beijing Research Institute of Science and Technology, Beijing 100089, China

**Keywords:** organotin compounds, dioctyltin, SERS, Raman

## Abstract

Organotin compounds (OTCs) are toxic pollutants threatening ecosystems and human health, among which dioctyltin (DOCT), widely used in skin-contact textiles, can induce immune dysfunction and metabolic disorders. Although DOCT levels in textiles are strictly regulated by international standards, traditional GC-MS suffers from cumbersome derivatization, unsatisfactory repeatability, and lengthy analysis, highlighting the urgent demand for a rapid and sensitive detection approach. Herein, we developed a fast SERS-based strategy for DOCT determination using size-optimized Au@Ag core–shell nanoparticles as the substrate, which offers simple pretreatment, high efficiency, good uniformity, and excellent reproducibility. The SERS spectra and functional group vibration modes of DOCT were elucidated by density functional theory (DFT) calculations combined with experimental validation, and the peak at 301 cm^−1^ was identified as the characteristic peak for quantitative analysis. After extractant optimization, the method achieved a low LOD of 0.1 μg/L in real textile samples, with recoveries ranging from 86% to 108% and good linearity from 0.1 to 1000 μg/L (*R*^2^ = 0.9804). This approach provides a reliable, high-sensitivity alternative for rapid monitoring of DOCT residues in textiles.

## 1. Introduction

Organotin compounds (OTCs) are metal–organic substances synthesized by the direct combination of tin and carbon [1]. These compounds have found extensive applications in various fields, including organic synthesis, catalysis, anticorrosion, and sterilization. They are commonly utilized as catalysts, heat stabilizers, agricultural pesticides, fungicides, and antifouling coatings [2,3,4,5]. However, organotin compounds are the only organometallic substances recognized as endocrine disruptors within ecosystems. Through biomagnification and transmission across food chain levels, these compounds pose significant risks to human health [6]. The toxicological effects of organotin compounds are multifaceted, impacting various physiological systems [7,8,9,10]. At the cellular level, organotin compounds interfere with oxidative phosphorylation, disrupting the cellular energy utilization process. This disruption leads to insufficient energy supply, impairing normal cellular metabolism and survival. In the immune system, these compounds induce thymic atrophy, impairing the normal function of the thymus and lymphatic system, thus weakening the body’s immune defense and altering cellular immunity. In the digestive system, organotin compounds can cause pancreatic damage, thereby affecting the secretion of digestive enzymes and nutrient metabolism. In the nervous system, they trigger central nervous system disturbances, including white matter edema in the brain, and interfere with normal neural signal transmission. In the endocrine system, organotin compounds inhibit hormone secretion, potentially leading to metabolic disorders such as diabetes and hyperlipidemia. Moreover, these compounds have been implicated as potential carcinogens, presenting an additional threat to human health [11,12,13,14]. Aside from their systemic effects, direct contact with organotin compounds can lead to irritation of human skin, the respiratory tract, or the cornea, resulting in discomfort [15].

Dioctyltin (DOCT) is widely utilized in the production of textiles that come into contact with the skin, particularly as heat stabilizers in high-temperature dyeing or heat-setting processes, as well as catalysts in the manufacturing of synthetic fibers such as polyester and polyurethane. Additionally, dioctyltin is commonly incorporated into close-fitting sportswear and protective clothing to impart antimicrobial, antimildew, and antiodor properties, effectively inhibiting bacterial and fungal growth and ensuring the cleanliness and hygiene of fabrics [16,17]. However, dioctyltin presents several potential health risks. Prolonged skin contact with textiles containing excessive amounts of dioctyltin may lead to contact dermatitis, characterized by symptoms such as redness, itching, and rashes, thus compromising skin health [18]. Beyond direct contact, dioctyltin can be absorbed through the skin and enter the body undetected, or it may adhere to the hands and subsequently be ingested via food, disrupting the normal function of the endocrine system and causing harm to the immune system [19]. However, there are relatively few studies on DOCT in textile samples. Therefore, it is also important to further investigate the detection of DOCT in textiles. The EU Organic Tin Compounds Directive (2009/425/EC) currently governs the use of organotin compounds within the European Union. This directive specifically restricts the use of organotin compounds, including DOCT compounds, and mandates that, as of 1 January 2012, textile products intended for public use or direct skin contact, as well as childcare and feminine hygiene articles, must not contain DOCT compounds with a tin content exceeding 0.1 wt%.

Various methods have been developed for the determination of organotin compounds [20,21,22,23,24,25,26], including gas chromatography–mass spectrometry (GC-MS) [27], high-performance liquid chromatography–mass spectrometry (HPLC-MS) [28], capillary electrophoresis [29], atomic absorption spectrometry (AAS) [30], and inductively coupled plasma–mass spectrometry (ICP-MS) [31]. Among these, GC-MS is the primary method used for detecting DOCT in textiles, with a detection limit as low as 30 µg/L.

While traditional detection methods for trace-level organotin compounds can be effective, they present several challenges. The derivatization process involved is complex and prone to errors, often being time-consuming and resulting in poor reproducibility. Recently, surface-enhanced Raman spectroscopy (SERS) has emerged as a promising alternative due to its high sensitivity, suppression of fluorescence background, simple sample preparation, rapid detection speed, and minimal sample volume requirements [32]. SERS holds significant potential for safety detection applications, particularly in organotin detection. The SERS technique integrates Raman spectroscopy with nanoscience, where the molecules to be detected are adsorbed onto or near the rough surface of a metal. Electromagnetic waves stimulate the metal’s surface plasmon resonance (SPR), leading to a significant enhancement of the electric field strength at the metal surface. As a result, molecules in close proximity to the metal experience a substantial enhancement of their Raman signals, often by several orders of magnitude [33,34,35,36,37,38,39]. This interaction greatly improves the detection sensitivity, thereby facilitating the analysis of trace amounts of target compounds. While SERS has been successfully applied to detect trisubstituted tin compounds in previous studies [40,41,42,43,44,45,46], there has been no research focused on the detection of DOCT.

In this study, we investigated the Raman spectra and vibrational modes of DOCT based on density functional theory (DFT) calculations. We synthesized and optimized gold–silver core–shell nanoparticles (Au@Ag NPs) as SERS-active substrates. The SERS response and reproducibility of DOCT on this nanostructure were examined. Furthermore, we optimized the extractant for DOCT in combination with real textile samples and established a rapid, sensitive, and reliable analytical method for determining DOCT in textile samples.

## 2. Materials and Methods

### 2.1. Materials and Reagents

Dioctyltin dichloride (C_16_H_34_Cl_2_Sn, 97%) was from Aladdin Reagent Co., Ltd. (Shanghai, China); methanol (CH_3_OH), ethanol (C_2_H_5_OH), *n*-butanol (C_4_H_9_OH), *n*-hexane (C_6_H_14_), sodium citrate (C_6_H_5_Na_3_O_7_·2H_2_O), silver nitrate (AgNO_3_), and tetrachloroauric acid (HAuCl_4_) were of analytical grade and were supplied by Sinopharm Group Chemical Reagent Co. (Shanghai, China).

### 2.2. Preparation of Au@Ag Nps

Au NPs (~26 nm) were synthesized via a modified Frens method [47] with sodium citrate reduction. A precleaned three-necked flask was charged with 100 mL DI water and 1 mL 1% chloroauric acid and heated to 140 °C. Upon boiling, 1.5 mL 1% trisodium citrate was added and stirred at 600 rpm for 15 min until wine-red, then cooled.

For Au@Ag NPs (~35 nm), 50 mL as-synthesized Au NPs were diluted to 100 mL, mixed with 1 mL sodium citrate, and heated to boil. A total of 0.5 mL 0.45% silver nitrate solution was added, stirred for 20 min, cooled, and centrifuged at 7200 rpm for 10 min. The pellet was resuspended in 50 μL DI water.

### 2.3. Characterization of the Physicochemical Properties of Au@Ag Nps

The physicochemical properties of Au@Ag NPs were characterized by ultraviolet–visible (UV-Vis) absorption spectroscopy, scanning electron microscopy (SEM), transmission electron microscopy (TEM), and energy spectrum analysis (EDS).

UV-Vis spectroscopy: UV-Vis spectra of Au and Au@Ag NP substrates were recorded using a U-3310 dual-beam spectrophotometer (Hitachi, Tokyo, Japan). Substrates were diluted via ultrasonic mixing with DI water, transferred to a quartz cuvette (≤2/3 volume), and scanned from 200 to 800 nm at 0.5 nm intervals.

SEM: NPs were diluted with DI water, drop-cast (6 µL) onto a wafer, and air-dried at 60 °C. Morphology and size were observed using a SU8010 SEM (Hitachi, Tokyo, Japan).

TEM/EDS: Centrifuged Au@Ag NPs were deposited on a copper mesh, air-dried, and imaged via a Spectra 300 STEM (Thermo Fisher Scientific, Waltham, MA, USA). TEM-EDS mapping characterized NP morphology, size, elemental distribution, and composition.

### 2.4. Theoretical Calculations of Organotin Raman Characteristics

DFT [48] calculations were performed using the Gaussian 16 software package. The B3LYP functional was employed for the theoretical calculation of organotin compounds [49]. A mixed basis set was used, with the 6-311++g(d,p) [50] basis set applied to the non-metallic atoms (C, H, Cl) and the Lanl2DZ [51] basis set used for the metallic atom (Sn).

For the structural optimization and gas-phase frequency calculation of DOCT, the B3LYP/gencp mixed basis set was employed—consistent with the one described above. The calculation procedure was as follows: first, the solute was converted from its pure state to the gaseous state and placed in a vacuum environment; subsequently, the gaseous solute was transferred from the vacuum into the solvent. After the completion of gas-phase frequency calculation, the vaporization free energy (ΔG__vap_) was obtained. On this basis, by modifying the “solvent=” keyword in the scrf model and incorporating solvents with different dielectric constants, the free energies of dioctyltin dichloride in four solvents were calculated, including a high-polarity solvent (methanol), medium-polarity solvents (*n*-butanol and ethanol), and a low-polarity solvent (*n*-hexane). The solvation free energy (ΔG__solv_) for each solvent was then acquired. Finally, the total dissolution free energy (ΔG__sol_) was derived by subtracting ΔG__vap_ from ΔG__solv_.

### 2.5. Normal Raman and SERS Measurements

Raman and SERS spectra were recorded using a Horiba LabRAM HR confocal micro-Raman system with a 532 nm laser (maximum power 100 mW). A 50× objective lens was adopted, with an estimated laser spot diameter of approximately 1440 nm. To avoid photothermal or photochemical degradation of DOCT or the Ag shell, 1% of the full power (≈1 mW) was employed during detection. As shown in Figure S3, time-dependent measurements verified the stability of the SERS signal. Acquisition parameters: 2 cm^−1^ spectral resolution, 10 s integration time, 2 accumulations.

Standard solution preparation: A 100 mg/L DOCT stock solution was prepared in ethanol and then diluted with DI water to obtain 1 ng/L–100 mg/L concentration gradients.

DOCT Raman spectra acquisition: A total of 1 g of DOCT solid was compressed under a coverslip, and Raman spectra were collected with 20 scans per test point (0–3500 cm^−1^) for average peak intensity. Data were processed using Origin 2022.

SERS measurement: A total of 2 µL of Au@Ag NPs was deposited on a silicon chip, followed by 6 µL of DOCT standard solution (10-fold concentration gradients). The chip was dried at 60 °C, and SERS spectra (200–3500 cm^−1^) were acquired at the central region of the silicon chip. Ten test points were collected per sample for average peak intensity. As shown in Figure S4, the uniformity of the Au@Ag NPs substrate was confirmed by SEM characterization. As shown in Figure S5, no interference from the blank solvent on the detection of DOCT was observed. Data analysis was performed using Origin 2022.

### 2.6. Textile Sample Testing

Commercially available 100% polyester fabric was cut into 5 mm × 5 mm pieces, weighed (0.5 g each), and precleaned: rinsed three times with water, methanol-soaked for 10 h, then washed and dried. Seven samples were spiked with 20 mL of 100 µg/L DOCT, sealed, stirred for 24 h, and solvent-evaporated. Extraction was performed using methanol, *n*-butanol, *n*-hexane, or methanol–ethanol mixtures (4:1, 2:1, 1:1, 1:2), followed by 30 min ultrasonic extraction at room temperature. Extracts were pretreated for SERS detection, and 200–500 cm^−1^ scans (parameters as previously described) were processed using Origin 2022.

Five additional polyester fabric samples were spiked with DOCT standards (100 µg/L–1 mg/L), stirred for 24 h, evaporated, and extracted with 20 mL of a methanol–ethanol (2:1) mixture via 30 min ultrasonication at room temperature. Extracts underwent SERS analysis (200–1500 cm^−1^, identical parameters), with spectra plotted using Origin 2022.

### 2.7. Experimental Data Analysis

The acquired spectra were analyzed using the Origin 2022 software. In this study, two main methods were used for data preprocessing: baseline correction and smoothing. We used iterative adaptive weighted penalized least squares (airPLS) to correct the baseline [52]. Baseline drift and background noise in spectral data can be effectively eliminated. For data smoothing, we adopted the Savitzky–Golay (SG) smoothing algorithm for signal smoothing and noise abatement. To evaluate the relationship between the concentration of the DOCT standard solution and the Raman intensity of its characteristic peaks in the corresponding sample, a simple linear regression method was used. This method was employed to determine if there was a linear relationship between the two variables. The coefficient of determination (*R*^2^) was used to assess the goodness of fit of the linear regression, indicating how well the regression line approximated the observed data. A higher *R*^2^ value suggests a better fit and a stronger linear relationship between concentration and Raman intensity.

## 3. Results and Discussions

### 3.1. Characterization of Au@Ag Nps

**Figure 1:**
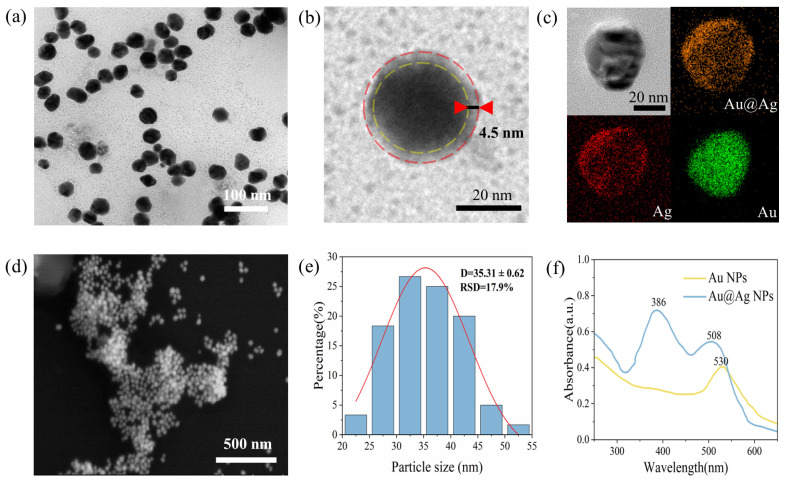
(**a**) TEM image of Au@Ag NPs; (**b**) the core–shell structure of Au@Ag NPs; (**c**) TEM-EDS mapping of Au@Ag NPs; (**d**) SEM image of Au@Ag NPs; (**e**) particle size distribution of Au@Ag NPs; (**f**) comparison of the UV–visible absorption spectra of Au@Ag NPs and Au NPs.

The Au@Ag NPs synthesized in this study combine the stability and uniformity of gold nanostructures with the strong Raman activity of silver nanostructures. The TEM image in Figure 1a reveals that the Au@Ag NPs have a spherical shape with homogeneous size and appearance. The TEM images in Figure 1b,c provide a more detailed view of the morphology and size of individual Au@Ag NPs, clearly illustrating the core–shell structure of the nanoparticles and the relatively uniform thickness distribution of the Ag shell. The EDS mapping reveals the elemental composition and distribution of the nanoparticles, confirming the uniform distribution of both gold and silver elements and the core–shell structure. This further confirms the successful synthesis of Au@Ag NPs. 

As shown in Figure 1d,e, the particle size distribution indicates that the Au@Ag NPs are predominantly in the range of 25–45 nm, with an average particle size (D) of 35.31 ± 0.62 nm and a relative standard deviation (RSD) of 17.9%. In contrast, Figure S1 shows the size of the Au NPs used in the synthesis, with the particle size primarily distributed between 23 and 29 nm, an average particle size of 26.59 ± 0.16 nm, and an RSD of 8.94%. The uniform size and homogeneity of the Au NPs validate the reliability of the synthesis method. The size distribution of the Au@Ag NPs further indicates that the stability of the synthesized particles is good, overcoming the issue of agglomeration commonly seen with Ag NPs. The uniform size distribution significantly improves the repeatability of SERS detection by reducing the tendency for Ag NPs to aggregate.

The UV absorption spectra of the synthesized Au NPs and Au@Ag NPs are presented in Figure 1f. The maximum absorption peak of Au NPs is observed at 530 nm, which corresponds to the SPR of Au NPs. Upon the addition of silver nitrate solution, Ag^+^ ions are reduced to Ag^0^ under the reducing effect of sodium citrate and gradually deposit onto the surface of the gold nuclei, leading to the formation of the core–shell structure of Au@Ag NPs. As a result, two distinct SPR absorption peaks at 387 nm and 509 nm emerge in the spectra, which are primarily attributed to the surface plasmon resonance of the silver shell and the gold core, respectively. The maximum absorption peak of Au NPs undergoes a blue shift, consistent with previous studies [53]. These observations confirm the successful synthesis of Au@Ag NPs and also demonstrate the excellent Ag shell activity of the substrate.

As shown in Figure S7, the FDTD simulation results of the electric field enhancement for different Ag shell thicknesses verify that 4.5 nm is the optimal thickness. Overall, the combination of particle size analysis and TEM images demonstrates that the average thickness of the Ag shells (4.5 nm) aligns with the experimental design and meets the core–shell size ratio for optimal performance of Au-Ag NPs reported in relevant studies [54], effectively optimizing the Raman signal enhancement effect of the substrate.

The significant SERS enhancement observed in this system mainly originates from the strong electromagnetic field enhancement induced by the SPR of the Ag shell. Meanwhile, the contribution of chemical enhancement cannot be ignored. The polar Sn–Cl bonds in DOCT allow Cl atoms to form stable coordination interactions with the Ag surface, which promotes the chemisorption of DOCT molecules onto the Ag shell. Such chemisorption rearranges the molecular energy levels and facilitates the photo-induced charge-transfer (PICT) process. Under laser excitation, electrons can transfer from the Fermi level of Ag to the unoccupied orbitals of the adsorbed molecules, forming charge-transfer states and further increasing the molecular polarizability and Raman intensity. Therefore, the overall SERS enhancement arises from the synergistic effect of electromagnetic enhancement and charge-transfer-based chemical enhancement, in which electromagnetic enhancement plays a dominant role.

The selective enhancement of different vibrational modes provides clear evidence for the adsorption geometry of DOCT on the Ag surface. According to the SERS surface selection rule, vibrational modes with components perpendicular to the metal surface are preferentially enhanced. In this work, the Sn–Cl stretching and C–C backbone stretching modes are significantly enhanced, while the C–H bending mode is relatively weak. This result indicates that DOCT molecules are anchored onto the Ag surface via Cl atoms, with the main molecular axis adopting a nearly vertical or highly tilted orientation. In this configuration, the Sn–Cl and C–C stretching vibrations have large components perpendicular to the substrate surface, leading to strong coupling with the enhanced electromagnetic field. In contrast, the C–H bending mode exhibits polarization changes mostly parallel to the surface, resulting in weaker enhancement. This adsorption geometry is consistent with the coordination interaction between Sn–Cl and Ag and provides a favorable pathway for charge-transfer enhancement.

### 3.2. Raman Spectroscopic Characterization of DOCT

Raman spectroscopy facilitates the characterization of molecular structures and chemical compositions by measuring vibrational modes inherent to covalent bonds. Each distinct functional group and its intermolecular interactions exhibit unique vibrational frequencies, which manifest as spectral peaks in Raman spectra. These vibrational signatures serve as chemical fingerprints, enabling spectroscopic differentiation of molecular species.

**Figure 2:**
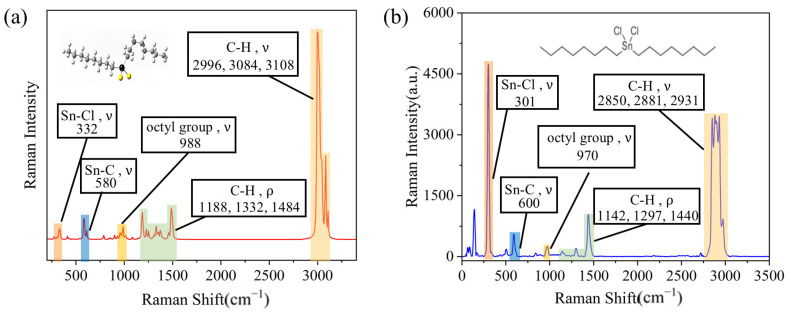
(**a**) DOCT Raman profile obtained from a study of DFT of pharmaceuticals. The black spheres represent Sn, the yellow spheres represent Cl, and the rest represent carbon chains. (**b**) DOCT Raman profile obtained from solid samples based on real measurements. ν: stretching vibration; ρ: bending vibration.

As depicted in Figure 2a, the theoretical Raman spectra of DOCT exhibit several distinct characteristic peaks at 332 cm^−1^, 580 cm^−1^, 988 cm^−1^, 1188 cm^−1^, 1332 cm^−1^, 1484 cm^−1^, 2996 cm^−1^, 3084 cm^−1^, and 3108 cm^−1^. Animated diagrams of functional group vibrations corresponding to Raman peaks have been provided in the Supplementary Materials, as shown in Table S1.

To analyze the Raman spectral features of DOCT, the measured Raman spectra shown in Figure 2b were divided into three distinct intervals within the spectral range of 0–3500 cm^−1^. By comparing the experimental data with the theoretical calculation results, the vibrational modes of the functional groups corresponding to the Raman peaks were confirmed, as shown in Table 1.

In the low-frequency region (0–400 cm^−1^), the most prominent characteristic peak in the Raman spectrum of DOCT corresponds to the stretching vibration of the Sn–Cl bond at 301 cm^−1^, which aligns with the theoretical calculation at 332 cm^−1^. This peak can be considered a qualitative characteristic feature of DOCT. Additionally, relative motions of the ligand atoms around the tin atom, including the bending vibrations of the Sn–C and Sn–Cl bonds (within the range of 100–200 cm^−1^), are observed. The peak at 140 cm^−1^ is likely associated with these bending vibrations.

In the mid-frequency region (400–500 cm^−1^), peaks corresponding to the stretching vibration of the Sn–C bond (600 cm^−1^), the stretching vibration of the C–C bond in the main chain of the octyl group (970 cm^−1^), and the bending vibration of the C–H bond in the alkyl chain (1142 cm^−1^, 1297 cm^−1^, and 1440 cm^−1^) are observed. These peaks closely correlate with the theoretical results at 580 cm^−1^, 988 cm^−1^, 1188 cm^−1^, 1332 cm^−1^, and 1484 cm^−1^.

In the high-frequency region (1500–3500 cm^−1^), stretching vibrations of the C–H bonds in the alkyl chains are prominent, with peaks at 2850 cm^−1^, 2881 cm^−1^, and 2931 cm^−1^. These vibrational modes, typically found in the high-frequency range, are strong and approximately correspond to the theoretical peaks at 2996 cm^−1^, 3084 cm^−1^, and 3108 cm^−1^.

By analyzing these characteristic peaks, comprehensive information regarding the molecular structure and bonding of DOCT can be obtained. In summary, as presented in Table S2, the Raman characteristic peaks of DOCT are classified as follows: in the low-frequency region (0–400 cm^−1^), the primary feature is the stretching vibration of the Sn–Cl bond; in the mid-frequency region (400–1500 cm^−1^), the key features include the stretching vibration of the Sn–C bond, the stretching vibration of the octyl C–C bond, and the bending vibration of the C–H bond; and in the high-frequency region (1500–3500 cm^−1^), the predominant feature is the stretching vibration of the C–H bond.

### 3.3. SERS Detection of DOCT Standard Solutions

Raman peaks are characterized by their Raman shifts and intensities in a Raman spectrum. The intensity of these peaks can be influenced by various factors, which may differ between samples, such as sample concentration, laser power stability, and laser wavelength. However, as long as the chemical bonds of the constituent molecules remain unchanged, their Raman shifts will remain consistent. To minimize the effects of irrelevant information and noise, preprocessing of the raw spectral data is necessary to ensure that the spectral intensity accurately reflects the concentration of DOCT. In this experiment, baseline correction and smoothing were applied to the obtained Raman spectra prior to data analysis. The height of the spectral bands, corresponding to the Raman intensities, was then used to represent the Raman information in relation to the DOCT concentration.

Figure S2 illustrates the results of Raman detection of a standard solution of DOCT at various gradient concentrations, utilizing core–shell Au@Ag NPs as the SERS substrate. From Figure S2, it is evident that the characteristic peaks near 301 cm^−1^ (Sn–Cl stretching vibration) and 970 cm^−1^ (octyl C–C stretching vibration) are more prominent in the SERS spectra. In contrast, the peaks associated with the C–H bending vibrations at 1142 cm^−1^, 1297 cm^−1^, and 1440 cm^−1^ are less pronounced, suggesting that the SERS substrate provides more significant enhancement for the stretching vibrations.

**Figure 3:**
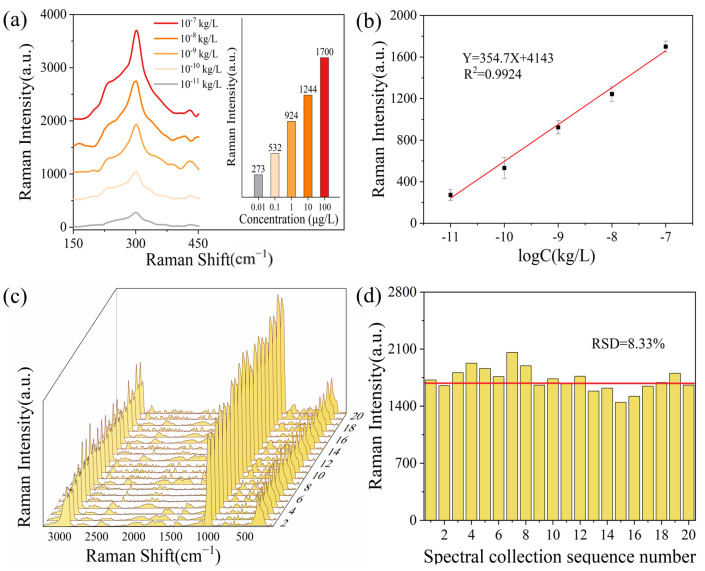
(**a**) DOCT standard solution gradient concentration SERS profile at 301 cm^−1^. The SERS spectra are displayed with a vertical offset of 500 counts for each concentration to avoid overlap and facilitate observation. (**b**) Raman peak intensity and linearity at 301 cm^−1^ of the SERS profile. (**c**) Waterfall of the SERS profile measured for different batches of Au@Ag NPs. (**d**) Column intensity distribution of 20 randomly selected points at 301 cm^−1^ of different batches of Au@Ag NPs. The red horizontal line represents the average value of the peak intensities.

Upon comparison of several key characteristic peaks, it is clear that the peak at 301 cm^−1^ is the most suitable marker for both qualitative and quantitative Raman analysis of DOCT, based on its linear relationship and the vibrational modes corresponding to functional groups. Figure 3a presents the Raman spectra at 301 cm^−1^ of the standard DOCT solution across a gradient concentration range of 0.01 µg/L to 100 µg/L. As the DOCT concentration decreases, the corresponding intensity of the Raman peak signals also diminishes. As shown in Figure 3b, a strong linear relationship is observed between the Raman intensity at 301 cm^−1^ and the DOCT concentration in the range of 10 ng/L to 100 µg/L. The linear equation is expressed as Y = 354.7X + 4143, with a correlation coefficient (*R*^2^) of 0.9924. This indicates that SERS is an effective method for the qualitative and quantitative determination of DOCT residues. 

In the application of SERS analysis, the stability of the substrate is a crucial factor affecting the accuracy and precision of the results. To verify the reproducibility of the Au@Ag NP substrates, five batches of Au@Ag NPs were prepared and subjected to multisite testing using DOCT standard solutions. This was done to ensure the good stability of the substrate. The concentration of the DOCT solution (100 µg/L) and the experimental conditions were kept consistent throughout the tests. As shown in Figure 3c,d, the Raman intensities at 301 cm^−1^ for the 20 measured values ranged from 1446 to 2060 a.u. The sample standard deviation was 143.6 a.u., with a mean value of 1724.2 a.u. and an RSD of 8.33%. The RSD being less than 20% indicates that the Au@Ag NPs substrates fabricated using the experimental method described in this paper exhibit good reproducibility. This low RSD [55] successfully verifies the reliability and consistency of the Au@Ag NP substrates for SERS applications.

### 3.4. Detection of DOCT in Textiles

Ultrasonic cavitation facilitates efficient release of DOCT from both the interior and surface binding sites of textile fibers. However, the extreme high-temperature and high-pressure environment of cavitation imposes strict requirements on extractants, making extractant selection critical to ultrasonic extraction efficiency. Extractant stability is pivotal to successful extraction: stable extractants under cavitation conditions resist decomposition, deterioration, or unintended reactions, ensuring consistent and reliable extraction. Furthermore, extractants with specific reactivity can engage in targeted chemical interactions with DOCT, enhancing extraction efficiency and selectivity—critical for isolating DOCT from complex matrices like textile fibers.

Extractants for ultrasonic extraction in SERS tests must meet key criteria, prioritized by their impact on testing, sample integrity, and practicality. Firstly, they should have low boiling points and high volatility, enabling easy removal via evaporation or drying to accelerate SERS testing. Additionally, they must minimize SERS spectral background interference to improve detection sensitivity. Furthermore, good compatibility with most textiles is required to avoid damaging textile structure or performance while preserving textile integrity during DOCT extraction. They should also be chemically stable at ambient temperature and pressure and unreactive with DOCT, ensuring DOCT’s chemical structure and properties remain intact for accurate extraction. Finally, when selecting commonly used organic solvents as extractants, high cost-effectiveness, ready availability, and low toxicity should be prioritized to ensure safe and efficient extraction. DOCT has a distinct dual polar–nonpolar molecular structure: one end contains chlorine atoms (defining its polar region), while the other features a long hydrophobic alkyl chain. Given this dual nature, careful extractant selection is necessary to optimize ultrasonic extraction efficiency and DOCT solubility.

**Figure 4:**
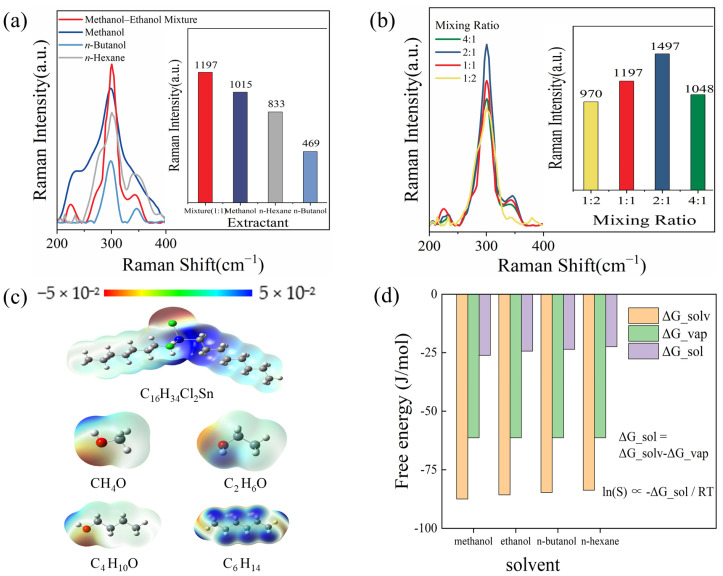
(**a**) Comparison diagrams of SERS performance extracted with different polar extractants; (**b**) comparison diagrams of SERS performance extracted with methanol–ethanol mixed solutions at different ratios; (**c**) charge distribution diagrams of DOCT and different polar extractants. The blue region represents positive electrostatic potential, the red region represents negative electrostatic potential, and the rest are neutral regions; (**d**) free energy of dissolution for DOCT with different polar extractants. ΔG_vap: free energy of vaporization; ΔG_solv: solvation free energy; ΔG_sol: free energy of dissolution; S: solubility; R: ideal gas constant; T: absolute temperature.

In this study, four extractants with varying polarities were used for ultrasonic extraction-SERS testing on textiles: high-polarity methanol, medium-polarity *n*-butanol, low-polarity *n*-hexane, and a methanol–ethanol mixture (with adjustable polarity). As shown in Figure 4a, by comparing the Raman intensities at 301 cm^−1^ of textiles spiked with DOCT at a concentration of 100 μg/L—following ultrasonic extraction in four different solvents and subsequent SERS measurement—it can be observed that the methanol–ethanol mixture exhibits the optimal effect. Furthermore, the effects of different volume ratios of methanol to ethanol in the mixture were tested, among which the ratio of methanol to ethanol at 2:1 showed the best performance, as presented in Figure 4b.

Theoretical calculations were performed to better analyze the results obtained from actual experiments. As shown in Figure 4c, the charge distribution map of DOCT exhibits a significant dual-region difference: due to the high electronegativity of chlorine atoms, the Sn–Cl polar bond forms a distinct negative charge accumulation zone, while the Sn atom carries a positive charge, creating a strongly polar core. The octyl alkyl chains (C_8_H_17_–) at both ends of the molecule show uniform charge distribution, as the electronegativities of carbon and hydrogen are comparable. This structure with coexisting polar and nonpolar domains dictates that the dissolution behavior of DOCT depends on the solvent’s ability to synergistically interact with these two structural domains. For the charge distribution maps of the four solvents, methanol (CH_3_OH), ethanol (C_2_H_5_OH), and *n*-butanol (C_4_H_9_OH) all contain strongly polar hydroxyl (–OH) groups. In these maps, oxygen atoms appear as red high-negative-charge regions, whereas hydrogen atoms in the hydroxyl groups are blue positive-charge regions; the nonpolar domain gradually expands with increasing alkyl chain length. The fourth solvent, *n*-hexane (C_6_H_14_), differs: its charge distribution is highly uniform and symmetric, with a larger positive-charge region.

The solubility of DOCT in various solvents derived from simulation calculations is presented in Figure 4d. The solvation free energy (ΔG_sol) data show that the ΔG_sol value of DOCT in methanol is −26.164 J·mol^−1^, which is higher than those in ethanol (−24.383 J·mol^−1^), *n*-butanol (−23.567 J·mol^−1^), and *n*-hexane (−22.429 J·mol^−1^). A lower ΔG_sol indicates stronger dissolution capacity; thus, theoretically, methanol exhibits the strongest dissolution capacity for DOCT.

Upon comparing experimental and theoretical results, the moderately polar n-butanol exhibits significantly lower extraction efficacy. Although *n*-butanol’s hydroxyl group can interact with DOCT’s polar region, steric hindrance from its long carbon chain hinders effective contact between hydroxyl groups and DOCT’s polar sites, potentially reducing hydrogen bonding strength and frequency. Additionally, *n*-butanol’s high viscosity impairs mass transfer efficiency, ultimately lowering DOCT extraction efficiency.

The low-polarity n-hexane mainly relies on dispersive van der Waals interactions. DOCT’s hydrophobic alkyl chains show good solubility in n-hexane due to their similar nonpolar nature, with the two components tightly bound via these interactions. However, n-hexane barely forms effective bonds or intermolecular interactions with DOCT’s polar region, limiting its overall extraction ability.

As a polar organic solvent, methanol contains highly polar hydroxyl groups (–OH). The electronegative chlorine atom (Cl) in DOCT imparts polarity to the region attached to the tin atom (Sn), enabling methanol to form hydrogen bonds with DOCT’s polar region. Van der Waals interactions between the two further promote solubility of DOCT’s polar region in methanol, but methanol’s high polarity limits its solubility for DOCT’s nonpolar moiety. Unlike methanol, ethanol is less polar but more molecularly flexible, with a longer carbon chain that enables more extensive interactions with DOCT’s alkyl chains via van der Waals interactions. The optimal methanol–ethanol ratio is 2:1; this mixture reduces methanol’s polarity while retaining solubilization capacity for both DOCT’s polar and nonpolar moieties. Notably, methanol’s high viscosity and surface tension weaken ultrasonic cavitation, whereas ethanol reduces the mixture’s viscosity and surface tension, improving ultrasonic extraction efficiency.

During extraction, the methanol–ethanol mixture synergizes with ultrasonic energy: methanol (high polarity) effectively interacts with DOCT’s polar region, while ethanol (higher molecular mobility) fills gaps to facilitate overall dissolution. Cooperative hydrogen bonding and van der Waals interactions between the solvents and DOCT enable efficient dissolution of the target compound.

Experimental results indicate that solvent screening should integrate practical mass transfer efficiency (e.g., n-butanol’s high viscosity and steric hindrance) and process conditions (e.g., methanol’s weakening of ultrasonic cavitation). The methanol–ethanol mixture emerges as the optimal extractant via a triple-advantage mechanism: “strong polar-region binding + easy nonpolar-region dispersion + ultrasonic-enhanced mass transfer”.

**Figure 5:**
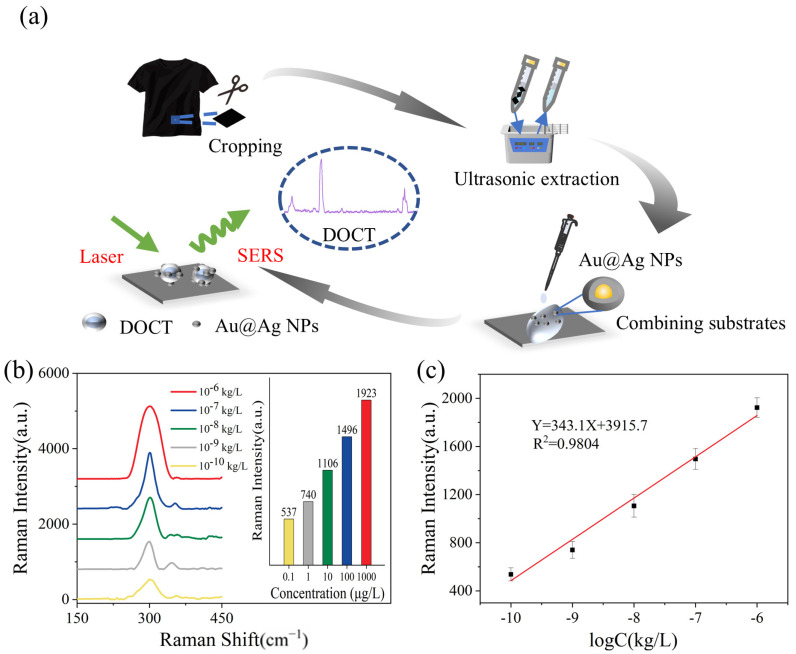
(**a**) Schematic diagram of the experimental procedure. The SERS spectra are displayed with a vertical offset of 800 counts for each concentration to avoid overlap and facilitate observation. (**b**) Gradient concentration SERS spectra of DOCT extracts in textiles. (**c**) Linear relationship plot between Raman intensity at 301 cm^−1^ and analyte concentration.

The Raman data obtained from DOCT in textiles—after ultrasonic extraction followed by SERS measurement—can be used for the quantitative analysis of DOCT. As shown in Figure S6, no characteristic peak is observed at 301 cm^−1^, indicating that matrix components from polyester textiles do not interfere with the SERS detection of DOCT. The schematic diagram of the experimental procedure is shown in Figure 5a. The SERS results for DOCT in textiles are presented in Figure 5b, where the spiked concentration of DOCT ranges from 100 ng/L to 1 mg/L. As the concentration of DOCT decreased, the signal intensity of the corresponding Raman characteristic peaks also diminished. A comparison of the SERS of the textile extract with those of the standard DOCT solution reveals that the characteristic Raman peak at 301 cm^−1^ corresponds to the one observed in the SERS spectrum of the standard DOCT solution, while they exhibit a difference in intensity. As shown in Figure 5c, a linear relationship between the Raman intensity at 301 cm^−1^ and the spiked concentration of DOCT in the textile extraction solution is evident within the concentration range of 100 ng/L to 1 mg/L. The linear equation is given by Y = 341.1X + 3915.7, with an *R*^2^ value of 0.9804 and a detection limit for DOCT of 0.1 µg/L. 

By comparing the linear relationship plots of Raman intensities at 301 cm^−1^ versus analyte concentrations for both the standard solutions and spiked samples obtained in the experiments, and using the “error propagation formula” and “t-distribution confidence interval”, the sample recovery rates of this method were calculated to be in the range of 86% to 108%. These results demonstrate that the SERS technique is capable of detecting DOCT in textiles, allowing for the rapid, sensitive, and reliable qualitative and quantitative analysis of DOCT residues in textile samples.

## 4. Conclusions

In this study, both theoretical and experimental Raman characteristics of DOCT were obtained through computational analysis and experimental data collection. We established the correlation between these Raman features and their corresponding vibrational modes of their functional groups. Additionally, we designed and synthesized Au@Ag NPs as substrates for SERS detection. EDS confirmed the elemental composition and core–shell structure of the substrates, while SEM and TEM images characterized the uniform dimensions and Ag shell thickness, which adhered to the design specifications. The fabricated substrates exhibited excellent SERS enhancement. The sensitivity and reproducibility of DOCT detection using SERS were thoroughly investigated, and a strong linear relationship was established between DOCT concentration and the intensity of its Raman characteristic peaks. Furthermore, an optimized extractant for ultrasonic extraction was identified to improve the extraction efficiency and solubility of DOCT from textiles.

Thus, a method for analyzing DOCT in textiles using the SERS technique was developed, integrating a suitable SERS substrate and the optimized ultrasonic extractant. In actual sample testing, trace amounts of DOCT were successfully detected, with a detection limit of 0.1 μg/L. The sample recovery rate ranged from 86% to 108%. This method holds strong potential for meeting international standards in the rapid quantitative detection of DOCT residues in textiles.

## Figures and Tables

**Table 1:** Table of corresponding vibration modes of DOCT’s Raman characteristic peak. ν: stretching vibration; ρ: bending vibration.

Raman Peak Corresponding to Vibrational Mode	Theoretically Calculated Raman Shifts (cm^−1^)	Experimentally Measured Raman Shifts (cm^−1^)	Difference in Raman Shifts (cm^−1^)
Sn–Cl, ν	332	301	31
Sn–C, ν	580	600	20
C–C of octyl group, ν	988	970	18
C–H, ρ	1188	1142	46
	1332	1297	35
	1484	1440	44
C–H, ν	2996	2850	146
	3084	2881	203
	3108	2931	177

## Data Availability

The original contributions presented in this study are included in the article/Supplementary Materials. Further inquiries can be directed to the corresponding authors.

## Supplementary Materials

The following supporting information can be downloaded at: https://www.mdpi.com/article/10.3390/s26061891/s1, Table S1. Table of corresponding vibration motion diagram of DOCT’s Raman characteristic peak. Table S2. DOCT Raman characteristic peaks and their corresponding vibrational modes. Figure S1. (a) SEM image of Au NPs (b) Particle size distribution of Au NPs. Figure S2. DOCT standard solution gradient concentration SERS profile. Figure S3. Time-dependent SERS spectra of DOCT recorded under 532 nm laser (1% power, ≈1 mW) for consecutive scans. The SERS spectra are displayed with a vertical offset of 500 counts for different time to avoid overlap and facilitate observation. Figure S4. Representative SEM image of the Au@Ag NPs assembled on the silicon substrate. Figure S5. SERS spectrum of ethanol solvent. Figure S6. SERS spectrum of the extract from blank polyester textile. Figure S7. (a) Simulated electric field distribution of Au@Ag NPs with different Ag shell thicknesses (b) Comparison of electric field intensity along the diameter in the cross-section of Au@Ag NPs with different Ag shell thicknesses (c) Comparison of the maximum electric field enhancement factor on the surface of Au@Ag NPs with different Ag shell thicknesses.
