# Supplementary material for: Rapid and Ultrasensitive Detection of Dioctyltin in Textiles Using Surface-Enhanced Raman Spectroscopy (SERS): Mechanistic Insights and Practical Applications
Source: Sensors (Basel). 2026 Mar 17;26(6):1891. doi: 10.3390/s26061891 (PMC13029820; doi:10.3390/s26061891)
Supplement: Supplementary file 1 [file sensors-26-01891-s001.zip › sensors-4170178-supplementary.pdf]

Rapid and Ultrasensitive Detection of Dioctyltin in Textiles Using Surface-Enhanced Raman Spectroscopy (SERS): Mechanistic Insights and Practical Applications

Supplementary Materials

Table S1 Table of corresponding vibration motion diagram of DOCT's Raman characteristic peak

Vibrational modes of functional groups	Raman shift (cm^{-1})	Corresponding vibrational mode
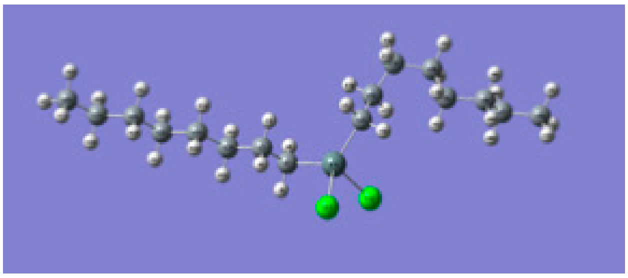	332	Sn-Cl, Telescopic vibration
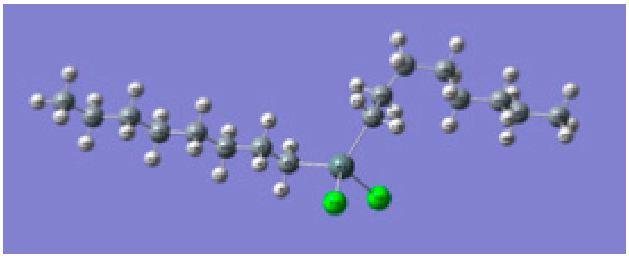	580	Sn-C, Telescopic vibration

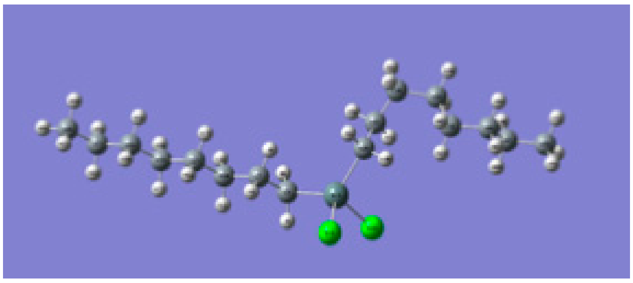	988	C-C of octyl group, Telescopic vibration
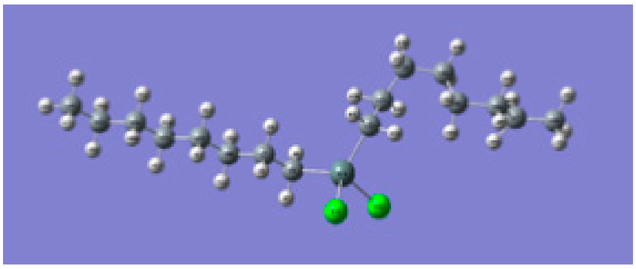	1188	C-H, bending vibration
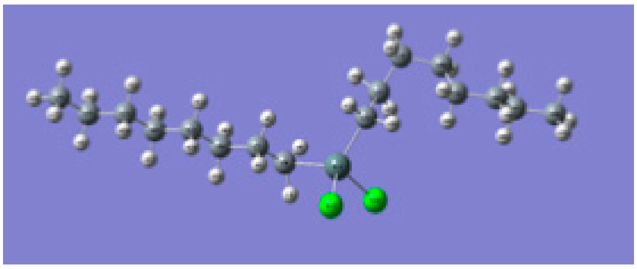	1332	
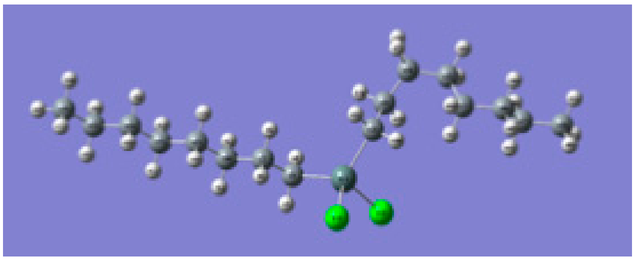	1484	
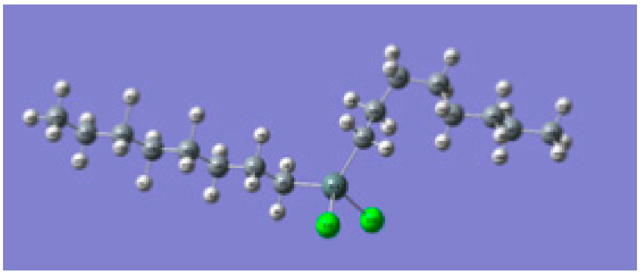	2996	C-H, Telescopic vibration

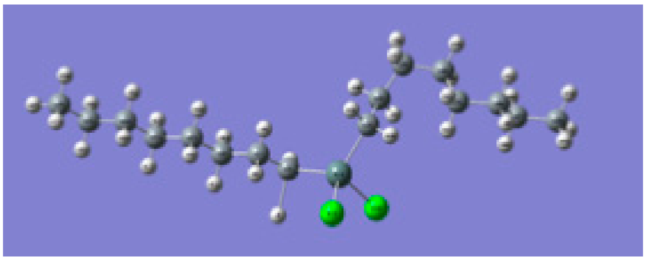	3084
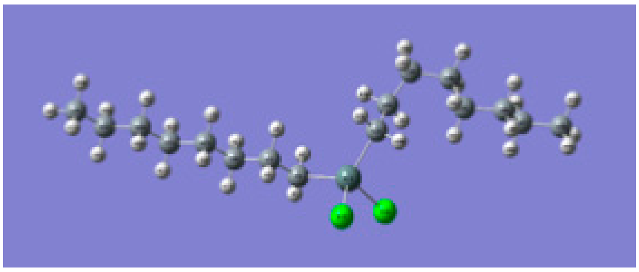	3108

Table S2. DOCT Raman characteristic peaks and their corresponding vibrational modes

Raman shift (cm^{-1})	Corresponding vibrational mode
301	Sn-Cl, stretching vibration
600	Sn-C, stretching vibration
970	C-C of octyl group, stretching vibration
1152	C-H,bending vibration
1297	
1436	
2847	C-H, stretching vibration
2881	
2918	

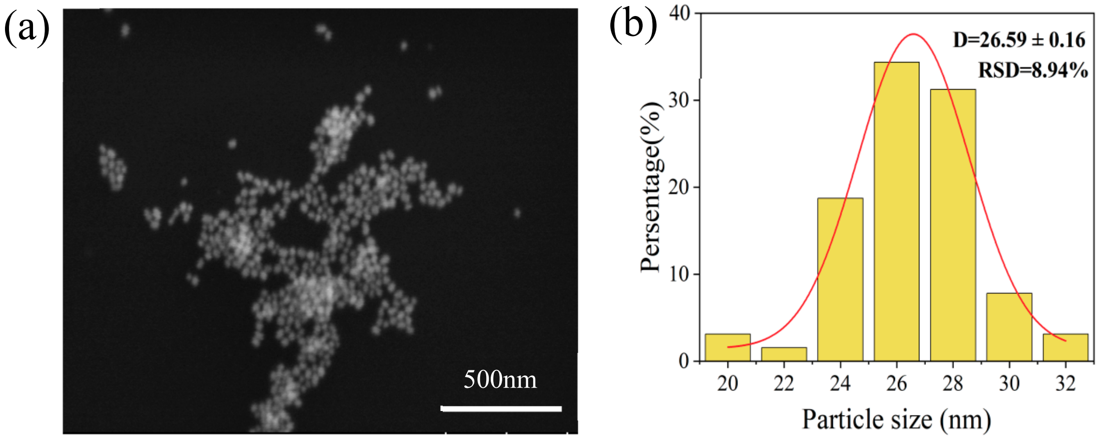

Fig. S1 (a) SEM image of Au NPs (b) Particle size distribution of Au NPs

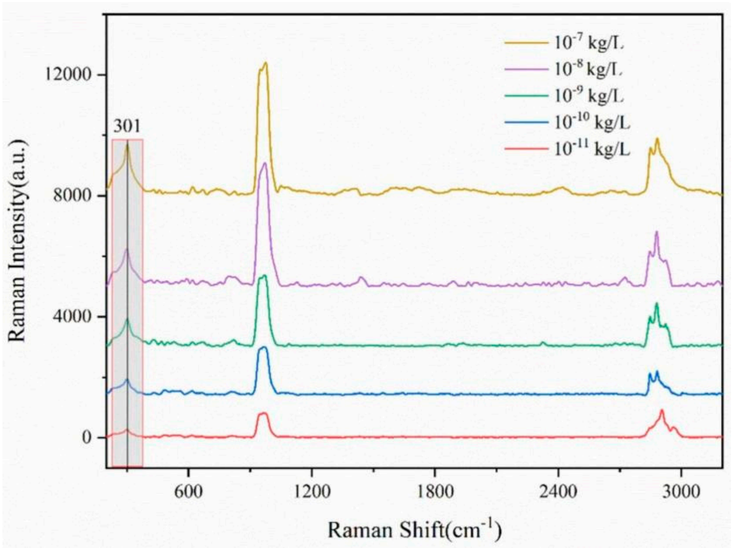

Fig. S2 DOCT standard solution gradient concentration SERS profile

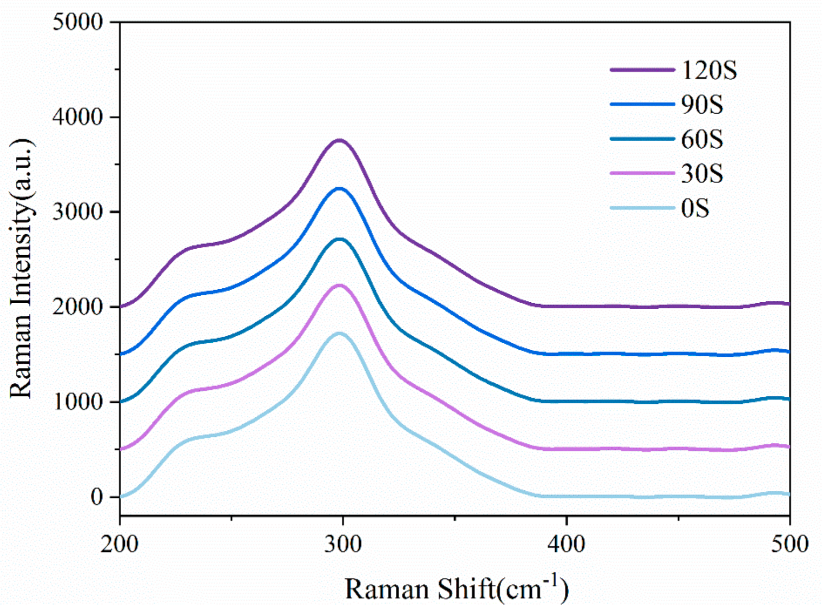

Fig. S3 Time-dependent SERS spectra of DOCT recorded under 532 nm laser (1% power, ≈1 mW) for consecutive scans. The SERS spectra are displayed with a vertical offset of 500 counts for different time to avoid overlap and facilitate observation.

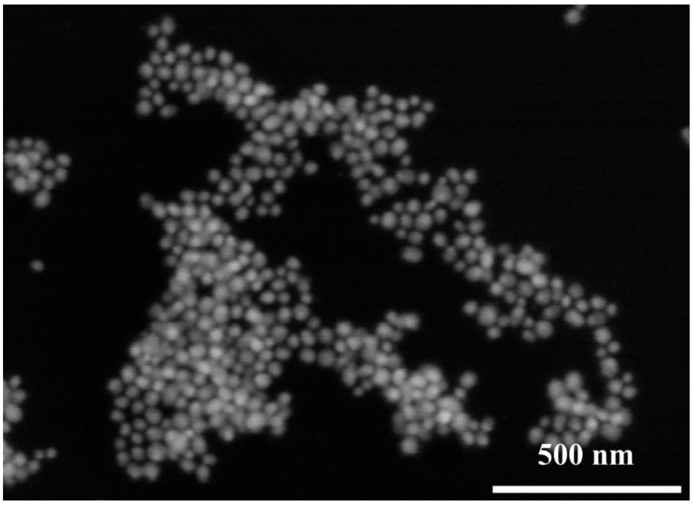

Fig. S4 Representative SEM image of the Au@Ag NPs assembled on the silicon substrate

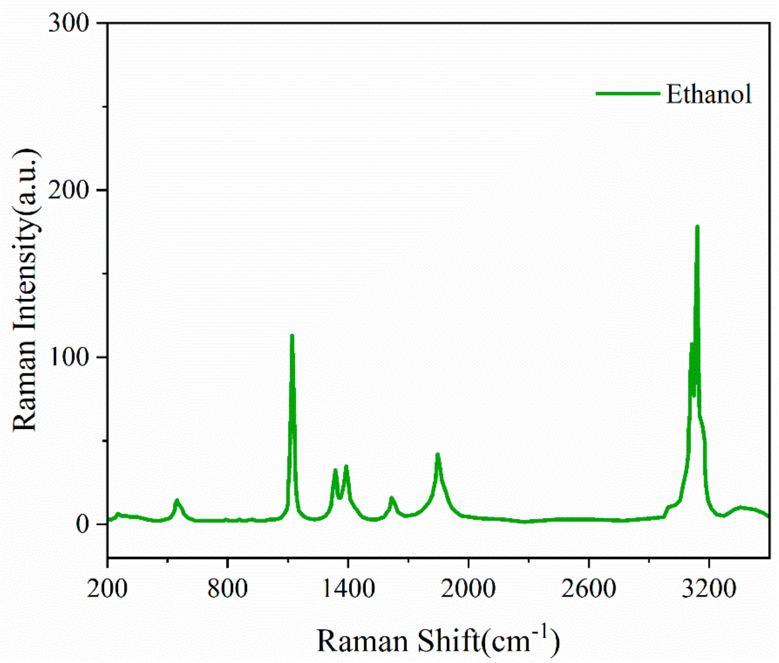

Fig. S5 SERS spectrum of ethanol solvent

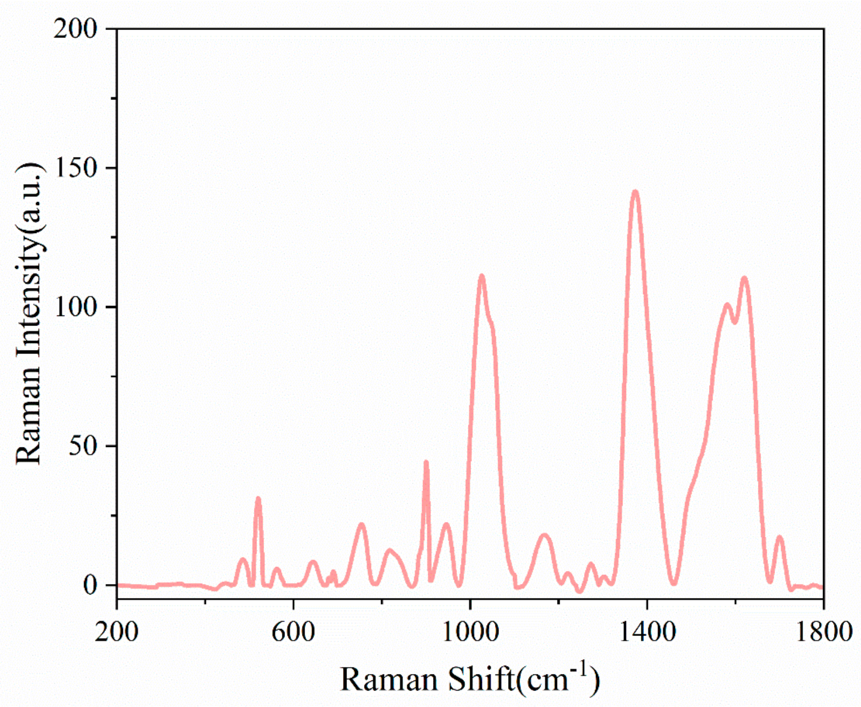

Fig. S6 SERS spectrum of the extract from blank polyester textile

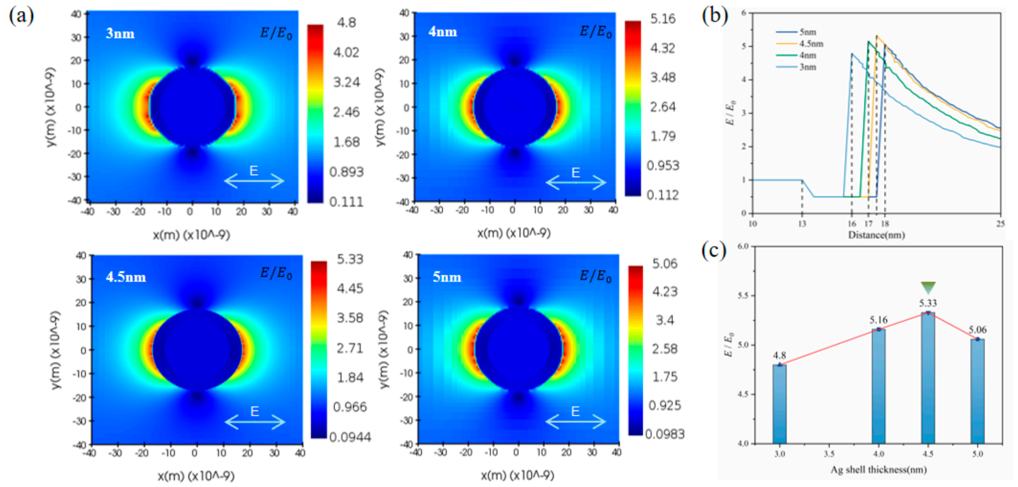

Fig. S7 (a) Simulated electric field distribution of Au@Ag NPs with different Ag shell thicknesses (b) Comparison of electric field intensity along the diameter in the cross-section of Au@Ag NPs with different Ag shell thicknesses (c) Comparison of the maximum electric field enhancement factor on the surface of Au@Ag NPs with different Ag shell thicknesses
